# Prognostic significance of endothelial dysfunction in patients undergoing percutaneous coronary intervention in the era of drug-eluting stents

**DOI:** 10.1186/s12872-015-0096-z

**Published:** 2015-09-23

**Authors:** Motoki Kubo, Toru Miyoshi, Hiroki Oe, Yuko Ohno, Kazufumi Nakamura, Hiroshi Ito

**Affiliations:** Department of Cardiovascular Medicine, Okayama University Graduate School of Medicine, Dentistry and Pharmaceutical Sciences, Okayama, Japan; Center of Ultrasonography, Okayama University Graduate School of Medicine, Dentistry and Pharmaceutical Sciences, Okayama, Japan

**Keywords:** Endothelial function, Stable angina pectoris, Restenosis, Percutaneous coronary intervention

## Abstract

**Background:**

Endothelial function is a prognostic predictor in patients undergoing percutaneous coronary intervention (PCI). However, in an era with widespread use of drug-eluting stents, the clinical relevance of endothelial dysfunction on restenosis in patients undergoing PCI has not been fully evaluated.

**Methods:**

This study included 80 patients with stable angina pectoris. Flow-mediated dilation (FMD) of the brachial artery was examined 1 week after PCI. Patients were retrospectively followed-up for an average of 21 months after PCI. The primary endpoints included cardiac death, nonfatal myocardial infarction, stroke, coronary revascularization, and critical limb ischemia.

**Results:**

A drug-eluting stent was used in 58 patients and a cardiovascular event was recorded in 34 patients during follow-up. The incidence of all cardiovascular diseases was significantly greater in the low FMD (median FMD <4.2 %) than the high FMD (median FMD ≥4.2 %) group (60 % vs. 25 %, *p* <0.01). Furthermore, the incidence of coronary revascularization was significantly higher in the low than the high FMD group (*p* = 0.02), while the incidence of in-stent restenosis did not differ between the two groups. Cox regression analysis showed that low FMD was an independent predictor of cardiovascular events (hazard ratio: 2.77, 95 % confidence interval: 1.23 to 6.19, *p* = 0.01).

**Conclusions:**

Impaired brachial artery FMD independently predicts long-term cardiovascular events after PCI in the era of drug-eluting stents.

## Background

Endothelial dysfunction leads to the initiation of atherosclerosis and is linked to many risk factors that predispose individuals to atherosclerosis [1–3]. Noninvasive ultrasound assessment of brachial artery flow-mediated dilation (FMD) has emerged as a method for studying nitric-oxide-dependent endothelial function [4]. Although reproducible FMD measurements require careful attention to training, technique, and analysis [5], previous studies have shown that FMD is a predictor of future cardiovascular events in populations with coronary risk factors [6, 7], and in patients with established coronary artery disease (CAD) [8, 9].

Percutaneous coronary intervention (PCI) with stenting is currently an effective and widespread treatment for patients with CAD. Although in-stent restenosis is a limitation of PCI, the use of drug-eluting stents (DES) has dramatically reduced the risk of restenosis [10]. Previous studies have shown that impaired FMD is a predictor of in-stent restenosis and cardiovascular events in patients undergoing PCI [11–14]. However, the use of DES modifies the association between endothelial function and in-stent restenosis because a drug released from the stent struts strongly suppresses the re-growth of endothelial cells onto stent struts [15]. Owing to the widespread use of DES in PCI, the association between impaired endothelial function and prognosis in patients undergoing PCI, including in-stent restenosis, needs to be re-evaluated.

We investigated whether early assessment of FMD predicts cardiovascular events, including in-stent restenosis, in patients undergoing PCI in the era of DES.

## Methods

### Study patients

This study enrolled 80 patients from among 138 consecutive patients with stable angina who were admitted to Okayama University Hospital for PCI and joined a cardiac rehabilitation program from August 2008 to February 2014. Patients who had angiographic documentation of organic stenosis of >70 % of at least one major coronary artery and had PCI successfully performed were eligible. Patients were excluded based on the presence of any of the following criteria: 1) acute coronary syndrome; 2) prior myocardial infarction; 3) history of stroke; 4) New York Heart Association functional classification ≥ III; 5) left main trunk disease; 6) left ventricular ejection fraction on echocardiography <40 %; 7) malignant disease; 8) chronic hepatic disease; 9) chronic inflammatory diseases; 10) chronic renal failure (serum creatinine levels >2.0 mg/dl); and 11) other serious systemic diseases. This study was approved by the institutional ethics committee of Okayama University Hospital. Written informed consent was provided by all of the patients before the study. The investigation conformed to the principles outlined in the Declaration of Helsinki.

### Study protocol

Measurement of FMD was performed in the morning after an overnight fast in the same manner at 1 week after PCI. All vasodilators were withdrawn 24 h before the FMD measurements. After PCI, all patients had individualized, optimized therapies, including medications and lifestyle changes, to reduce risk factors for CAD according to the American College of Cardiology/American Heart Association guidelines [16]. Levels of serum lipids, hemoglobin A1c, malondialdehyde-modified low-density lipoprotein (LDL) cholesterol, C-reactive protein, and adiponectin were measured, as described previously [17]. Patients were then retrospectively followed after PCI.

### PCI

PCI was performed with conventional techniques by the femoral or radial approach under systemic heparinization, and oral administration of aspirin and ticlopidine. The stent type and inflation pressure were chosen at the discretion of the physicians, who were blinded to the study protocol and the data regarding FMD. Procedural success was defined as reduction of stenosis to <30 % residual narrowing, with improvement of ischemic symptoms and without major in-hospital complications, such as death, emergency bypass surgery, or myocardial infarction (defined as >5 times increase in cardiac troponin T levels). After PCI, patients received aspirin (100 mg/day) indefinitely and ticlopidine (200 mg/day) or clopidogrel (75 mg/day) for at least 9 months. Original stented target lesion revascularization was defined as repeated PCI, and was performed in the presence of in-stent restenosis and any symptoms or objective signs of myocardial ischemia.

### Measurements of FMD in the brachial artery

FMD was assessed as a parameter of vasodilation according to the guidelines for ultrasound assessment [4]. Using a 10-MHz linear-array transducer probe (Unex Company Ltd., Nagoya, Japan), longitudinal images of the brachial artery at baseline were recorded with a stereotactic arm, and measurements of the arterial diameter were made after supine rest for ≥5 min. The diameter of the artery was measured, then suprasystolic compression (50 mmHg higher than systolic blood pressure) was performed at the right forearm for 5 min. Measurements of the arterial diameter were made continuously from 30 s after cuff release. Maximum vasodilation was then evaluated from the change in arterial diameter after release of occlusion. An experienced technician blinded to the clinical data of the study participants measured FMD and intra- and inter-observer correlation coefficients were high (>0.9) [18].

### Follow-up study

The primary endpoints included cardiac death, nonfatal myocardial infarction, coronary revascularization, critical limb ischemia, and stroke. The time to the first primary endpoint was evaluated retrospectively. The definition and assessment of endpoints were based on the statement from the American College of Cardiology/American Heart Association Task Force [19]. Myocardial infarction was defined as type 1 or type 2 myocardial infarction according to the Third Universal Definition of Myocardial Infarction [20]. All elective coronary revascularizations were undertaken only if the invasive fractional flow reserve of a coronary lesion was 0.80 or less [21]. Stroke included both ischemic and hemorrhagic types. Peripheral vascular intervention was documented in a Report of the American College of Cardiology/American Heart Association Task Force on Clinical Data Standards [20]. Critical limb ischemia needs emergency vascular intervention. Therefore, we included critical limb ischemia as a cardiovascular endpoint in this study. Data regarding primary and secondary outcomes were carefully collected from clinical charts and the diagnosis was confirmed by an investigator who was blinded to FMD data.

### Statistical analysis

Data are expressed as the mean ± standard deviation or number (percentage). The frequencies and continuous values between the two groups of patients were compared using the chi-square test and the Student’s t test, respectively. Kaplan–Meier analysis of event-free survival during follow-up was performed on the basis of the cut-off value of FMD. The association of FMD with future events was assessed by Cox proportional hazards analysis. The data were initially analyzed using a univariate model with covariates, including FMD and other potential confounders that were significantly different between patients with and without events. Multivariate Cox proportional hazards analysis was then applied using covariates that showed *p* < 0.1 in the univariate Cox proportional hazards analysis. Based on previous studies [11–14], we estimated that the incidence of cardiovascular events, including restenosis, in this study would be lower than that in previous studies because of the high use of DES. With an estimated event rate in patients with low versus high FMD of 30 % versus 4 %, respectively, a population of 80 patients would be needed to detect this difference with α = 0.05 and a power of 0.80. *p* < 0.05 was considered statistically significant. Statistical analysis was performed using SPSS 17.0 for Windows (SPSS Inc., Chicago, IL, USA).

## Results

**Fig. 1:**
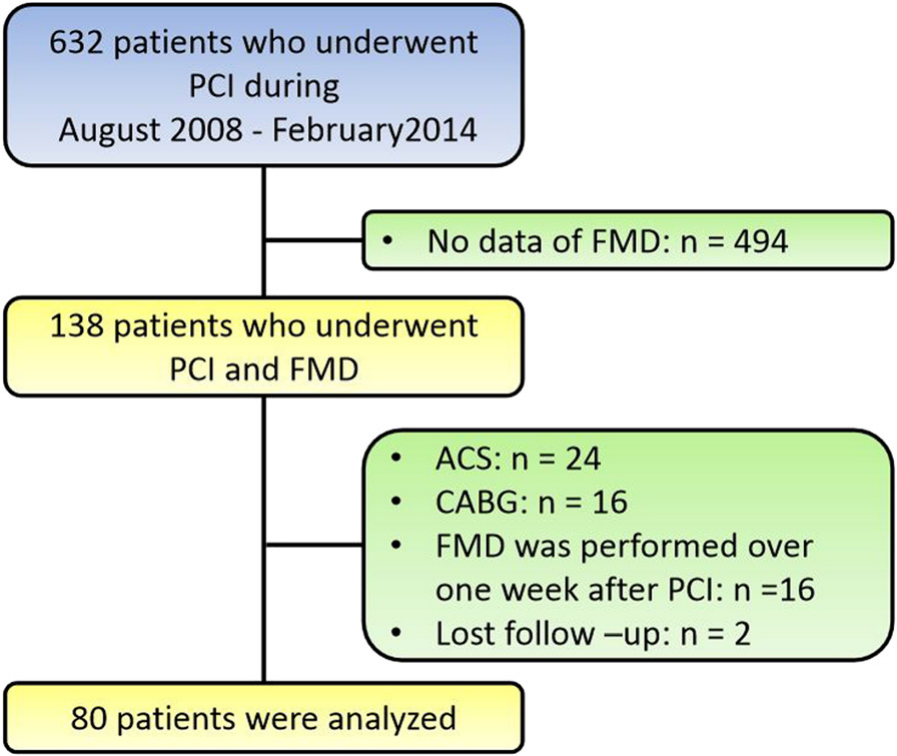
Flow diagram showing patients’ acceptance into the study

A flow diagram of this study is shown in Fig. 1. Of 632 patients who underwent PCI from August 2008 to February 2014, we excluded 494 patients without FMD data. Of the 138 remaining patients, 58 patients were excluded because of acute coronary syndrome (*n* = 24), coronary artery bypass graft (*n* = 16), FMD measured over 1 week after PCI (*n* = 16), and lost to follow-up (*n* = 2). Finally, 80 patients were analyzed.

The clinical characteristics of patients with and without an event are shown in Table 1. In this study, 56.3 % of patients had diabetes mellitus and 43.8 % had renal insufficiency. All of the patients were divided into two groups: the high FMD group (FMD ≥4.2 %, *n* = 40) and the low FMD group (FMD <4.2 %, *n* = 40), according to the median value of FMD. Patients with low FMD had lower high-density lipoprotein (HDL) cholesterol levels than those with high FMD (*p* = 0.04). Patients with low FMD tended to have greater body mass index and higher triglyceride levels than those with high FMD. There was no difference in prescription rates for angiotensin-converting enzyme (ACE) inhibitors and angiotensin II receptor blockers (ARBs) between the two FMD groups. Additionally, there was no difference in the type of ACE inhibitors between the two groups. There was also no difference in the type of statins between the two groups.

**Table 1:** Clinical characteristics in patients with FMD <4.2 % and ≥4.2 %

	FMD <4.2 % (*n* = 40 )	FMD ≥ 4.2 % (*n* = 40)	p
Age (years)	69.5 ± 7.1	69.4 ± 7.3	0.96
Female gender, n (%)	34 (85.0)	30 (75.0)	0.26
Body mass index	25.1 ± 3.4	23.6 ± 3.3	0.06
Diabetes mellitus, n (%)	23 (57.5)	22 (55.0)	0.82
Hypertension, n (%)	27 (65.7)	27 (67.5)	1.00
Dyslipidemia, n (%)	22 (55.0)	31 (77.5)	0.03
Current smoking, n (%)	10 (25.0)	11 (27.5)	0.80
Chronic renal insufficiency, n (%)	16 (40.0)	19 (47.5)	0.50
Previous PCI, n (%)	5 (12.5)	10 (25.0)	0.15
Previous myocardial infarction, n (%)	2 (5.0)	2 (5.0)	1.00
HDL cholesterol (mg/dl)	43.8 ± 11.2	49.3 ± 12.3	0.04
LDL cholesterol (mg/dl)	87.8 ± 28.1	88.1 ± 24.6	0.96
Triglycerides (mg/dl)	142.9 ± 73.6	113.7 ± 61.6	0.06
MDA-LDL cholesterol	97.9 ± 36.8 (*n* = 33)	82.2 ± 32.0 (*n* = 27)	0.62
Adiponectin	11.3 ± 5.2	12.0 ± 5.3	0.90
Medications			
Aspirin, n (%)	40 (100)	38 (95.0)	0.15
Clopidogrel, n (%)	35 (87.5)	33 (82.5)	0.53
ACE inhibitor/ARB, n (%)	35 (87.5)	30 (75.0)	0.15
ARB, n (%)	31 (77.5)	25 (62.5)	0.22
ACE inhibitors, n (%)	4 (10.0)	5 (12.5)	0.99
Perindopril	2 (5.0)	1 (2.5)	0.99
Imidapril	2 (5.0)	4 (10.0)	0.67
Statins, n (%)	36 (90.0)	36 (90.0)	1.00
Atorvastatin	4 (10.0)	7 (17.5)	0.52
Rosuvastatin	18 (45.0)	16 (40.0)	0.82
Pitavastaitn	14 (35.0)	13 (32.5)	0.99
β-blockers, n (%)	21 (52.5)	25(62.5)	0.37

Data are expressed as mean ± SD or number (percentage)

*FMD* Flow-mediated dilation, *PCI* Percutaneous coronary intervention, *HDL* High-density lipoprotein, *LDL* low-density lipoprotein, *MDA-LDL* Malondialdehyde-modified low-density lipoprotein, *ACE* Angiotensin-converting enzyme, *ARB* Angiotensin II receptor blocker

Procedural features of PCI are shown in Table 2. The percentage of the left anterior descending coronary artery as a target vessel artery was greater in the high FMD group than the low FMD group. The use of DES was 68 % in the low FMD group and 78 % in the high FMD group (*p* = 0.32). There were no differences in PCI-related features, such as lesion type, number of stents per lesion, stent diameter, stent length, and stent deployment pressure between the low and high FMD groups.

**Table 2:** Procedural features in patients with FMD <4.2 % and ≥4.2 %

	FMD <4.2 % (*n* = 40 )	FMD ≥ 4.2 % (*n* = 40 )	p
Target vessel artery	1.08 ± 0.27 (43 lesions)	1.23 ± 0.53 (49 lesions)	0.12
Left mail trunk, n (%)	3 (7.0)	5 (10.2)	0.46
Left anterior descending, n (%)	12 (27.9)	25 (51.0)	<0.01
Left circumflex, n (%)	16 (37.2)	12 (24.5)	0.35
Right coronary disease, n (%)	12 (27.9)	7 (14.3)	0.19
Multivessel coronary disease, n (%)	19 (47.5)	20 (50.0)	0.82
Lesion type B/C2, n (%)	40 (100)	39 (97.5)	0.31
Number of stets per lesion, n (%)	1.3 ± 0.6	1.3 ± 0.7	0.73
Stent diameter (mm)	3.03 ± 0.55	2.85 ± 0.51	0.13
Total stent length (mm)	22.4 ± 14.2	28.3 ± 16.9	0.09
Use of drug eluting stent (%)	27 (67.5)	31 (77.5)	0.32
Stent deployment pressure (atm)	18.3 ± 4.3	18.3 ± 4.5	0.98

**Fig. 2:**
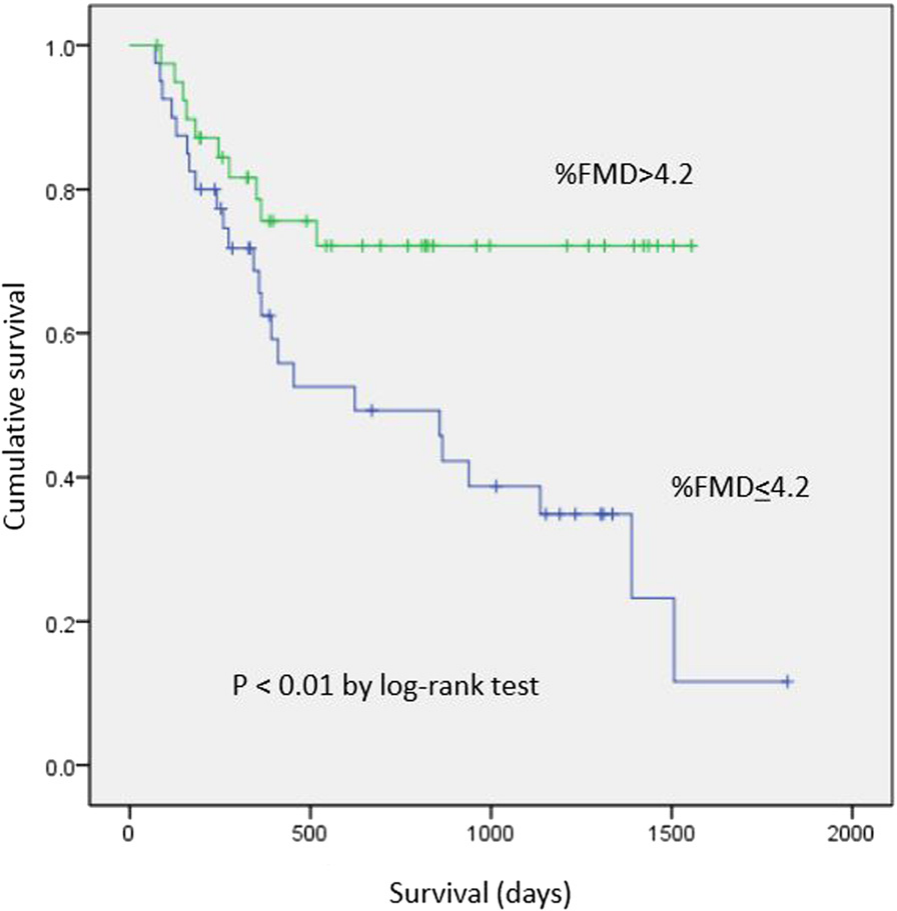
Kaplan–Meier survival curves for cardiovascular events in patients with FMD <4.2 % and ≥4.2 %

Patients were retrospectively analyzed for a mean of 21.4 ± 15.9 months after PCI. Table 3 shows the number of cardiovascular disease events when patients were dichotomously categorized as having low FMD or high FMD. During this follow-up period, 34 patients had a cardiovascular event, including cardiac death (*n* = 1), coronary revascularization (*n* = 28), critical limb ischemia (*n* = 1), or ischemic stroke (*n* = 4). Of 19 patients undergoing coronary revascularization due to new lesions, two patients in the low FMD group were hospitalized for unstable angina, but were not diagnosed as having myocardial infarction. PCI for new lesions in the low FMD group was performed significantly more frequently than that in the high FMD group, whereas target lesion revascularization was not different between the groups. FMD in patients with cardiovascular events (*n* = 34) was significantly lower than that in patients without cardiovascular events (*n* = 46) (3.4 ± 1.2 vs 5.1 ± 1.8, *p* < 0.01). There was no difference in the diameter of the brachial artery at baseline and after administration of nitroglycerin between patients with and without cardiovascular disease. The event-free survival curves are shown in Fig. 2. Patients in the low FMD group had significantly more events than those in the high FMD group (*p* < 0.01). In the Cox proportional hazards model including β-blockers and FMD, low FMD (<4.2 %) remained an independent predictor of cardiovascular events (Table 4).

**Table 3:** Cardiovascular events according to FMD

	FMD <4.2 % (*n* = 40)	FMD >4.2 % (*n* = 40)	p
Cardiac death, n (%)	1 (2.5)	0 (0)	0.31
Non-fatal myocardial infarction, n (%)	0 (0)	0 (0)	
Stroke, n (%)	3 (7.5)	1 (2.5)	0.31
Revascularization, n (%)	19 (47.5)	9 (22.5)	0.02
In stent restenosis, n (%)	5 (12.5)	4 (10.0)	0.72
New lesion, n (%)	14 (35.0)	5 (12.5)	0.02
Critical limb ischemia, n (%)	1 (2.5)	0 (0)	0.31
Total, n (%)	24 (60.0)	10 (25.0)	<0.01

**Table 4:** Univariate and multivariate Cox proportional hazards analyses of risk factors for cardiovascular events

	Univariate analysis			Multivariate analysis		
	Relative risk	95 % Confidential interval	p	Relative risk	95 % Confidential interval	p
Age >60 years	0.83	0.29–2.39	0.74			
Male	1.07	0.44–2.60	0.88			
Diabetes mellitus	1.53	0.76–3.062	0.23			
Hypertension	0.95	0.45–2.00	0.89			
Dyslipidemia	1.10	0.52–2.31	0.81			
Current smoking	1.32	0.64–2.71	0.45			
Chronic renal insufficiency	0.62	0.31–1.27	0.19			
Previous PCI	0.75	0.290–1.95	0.55			
Previous myocardial infarction	0.04	0.00–11.78	0.27			
LAD vs LCx/RCA	1.03	0.53–2.02	0.93			
Stent diameter <3.0 mm	1.33	0.66–2.67	0.43			
Stented segment length > 15mm	1.00	0.49–2.06	1.00			
Aspirin	21.14	0.00–159472.77	0.50			
Clopidogrel	1.38	0.48–3.93	0.55			
ACE inhibitor / ARB	0.98	0.42–2.26	0.98			
Statins	1.28	0.30–5.37	0.74			
β-blockers	0.50	0.25–1.00	0.05	0.56	0.28–1.13	0.11
FMD <4.2 %	2.60	1.24–5.46	0.01	2.40	1.14–5.06	0.02

*PCI* Percutaneous coronary intervention, *LAD* Left anterior descending coronary artery, *LCx* Left circumflex coronary artery, *RCA* Right coronary artery, *ACE* Angiotensin-converting enzyme, *ARB* Angiotensin II receptor blocker, *FMD* Flow-mediated dilation

## Discussion

This study shows that impairment of brachial artery FMD is an independent predictor of cardiovascular events, especially revascularization; however, the brachial artery FMD did not predict in-stent restenosis. Our findings suggest that early evaluation of endothelial function of the brachial artery after PCI can predict cardiovascular events, even in the era of DES.

Our results are in line with previous findings that impaired FMD of the brachial artery is associated with adverse outcomes in patients undergoing coronary stent implantation [8, 11–14, 22], but our study has some differences. In the studies by Patti et al. [14] and Munk et al. [13], the authors reported that impairment of FMD at 30 days after PCI predicted in-stent restenosis in patients with stable CAD at follow-up, while the use of DES was 3 % and 21 %, respectively. However, in our study, almost 70 % of patients were treated with DES. The mechanisms involved in in-stent restenosis include platelet and inflammatory cell activation due to procedural vascular injury, leucocyte adherence, smooth muscle cell proliferation, and extracellular matrix synthesis [23]. Endothelial function affects the association of these factors with in-stent restenosis of bare metal stents. However, in the case of DES, the effect of endothelial function on restenosis may be decreased because of strong suppressive effects of coated drugs on the stents.

Endothelial dysfunction has been proposed to be a “barometer” of vascular conditions that integrate the overall effects of risk factors and fundamental defense mechanisms [24]. Therefore, endothelial function determined by FMD in the brachial artery could be associated with the risk of new coronary lesions. In line with this concept, our study showed a significant association of FMD with coronary revascularization in new lesions, but not with in-stent restenosis. Late and very late stent thrombosis is a serious issue associated with DES [25]. Endothelial dysfunction is significantly associated with residual platelet aggregability after dual antiplatelet therapy [26]. The observation period of this study was not sufficient to evaluate late and very late stent thrombosis of DES. The association between stent thrombosis and systemic endothelial dysfunction needs to be investigated in a future large study.

Endothelial function reflects the atherosclerotic risk burden at the time of its measurement [27–30]. Our study showed that the low FMD group had lower HDL cholesterol levels and tended to have higher triglyceride levels than the high FMD group, while LDL cholesterol levels were comparable between the two groups. Almost 90 % of our population used statins; therefore, these factors indicated the exact residual risks of CAD. Low HDL cholesterol and high triglyceride levels may contribute to an increase in small dense LDL [31]. Small dense LDL is a highly atherogenic lipoprotein, which affects endothelial function [32, 33]. However, endothelial function is changed by modification of the atherosclerotic risk burden [34, 35]. Our previous study showed that a reduction in triglyceride levels by ezetimibe improved FMD in patients with CAD [36]. In terms of risk factor management, aggressive interventions for residual risks are needed to improve endothelial function, leading to an improvement in the prognosis of patients with CAD.

Endothelial function is affected by several medications. In terms of inhibition of the renin-angiotensin system, a significant difference has been observed between ARBs and ACE inhibitors [37, 38]; some studies have also suggested a difference between different ACE inhibitors [39]. In this study, no difference was observed in the prescription rates of ARBs, ACE inhibitors, or the type of ACE inhibitors between the low and high FMD groups. Statins also improve endothelial function; however, there may be a difference in the effect of different types of statins [40]. We have checked the type of statins used by the study participants, and no difference in the type of statins was observed between the two FMD groups. Thus, the use of these medications is unlikely to have affected the findings of the current study; however, long-term treatment with ACE inhibitors and statins may affect clinical outcome by pleiotropic effects beyond the influence of endothelial function.

Several studies have reported that impairment of endothelial vasomotor function has an adverse effect on clinical outcome in patients with CAD [8, 11–14]. However, there is currently no general agreement on the cut-off value for FMD. Therefore, the application of FMD in clinical practice is difficult. Currently, a large, multicenter prospective study is underway to determine normal values and cut-off values for FMD in the brachial artery, and to assess clinical outcomes [41]. This study will provide important evidence for the usefulness of FMD measurements in the risk stratification for cardiovascular disease.

Recently, use of a bioresorbable vascular scaffold (BVS) has been reported [42, 43]. The implantation of a BVS is a new approach that provides transient vessel mechanical support with drug delivery capability, potentially without permanent metallic implantations. In the process of bioresorption of the polymeric scaffold, endothelial function is important for early and appropriate covering and replacement of the scaffold by endothelial cells and extracellular matrix. Further data relating to the impact of endothelial function on the prognosis of patients with BVS implantation are eagerly awaited.

### Limitations

First, this study is preliminary and considerably limited by the small number of study patients. A large prospective trial is required to determine the precise role of systemic endothelial function in the pathogenesis of coronary atherosclerosis. Second, there were relatively few clinical events compared with the numerous variables tested in the multivariate model, resulting in large confidence intervals.

## Conclusions

This study shows that impaired brachial artery FMD is an independent determinant of cardiovascular events in the era of DES. In contrast to previous studies, FMD was not a predictor of in-stent restenosis in this study. Our results suggest that early assessment of endothelial function by brachial artery FMD may represent a useful screening tool for risk stratification of future cardiovascular events after PCI using DES. Further large prospective studies are required to evaluate the clinical utility of a brachial artery FMD method in patients with stable angina undergoing PCI.

## Abbreviation

ACE inhibitor: Angiotensin-converting enzyme inhibitor
ARB: Angiotensin II receptor blocker
BVS: Bioresorbable vascular scaffold
CAD: Coronary artery disease
DES: Drug-eluting stent
FMD: Flow-mediated dilation
HDL: High-density lipoprotein
LDL: Low-density lipoprotein
PCI: Percutaneous coronary intervention
